# Visual Navigation of Caged Chicken Coop Inspection Robot Based on Road Features

**DOI:** 10.3390/ani14172515

**Published:** 2024-08-29

**Authors:** Hongfeng Deng, Tiemin Zhang, Kan Li, Jikang Yang

**Affiliations:** 1 State Key Laboratory of Swine and Poultry Breeding Industry, South China Agricultural University, Guangzhou 510642, China; redmaple-deng@outlook.com (H.D.); sparrowlk65535@gmail.com (K.L.); yjkscau@gmail.com (J.Y.); 2 College of Engineering, South China Agricultural University, Guangzhou 510642, China; 3 National Engineering Research Center for Breeding Swine Industry, Guangzhou 510642, China

**Keywords:** cage chicken coop, inspection robot, visual navigation, road features

## Abstract

**Simple Summary:**

In the process of large-scale cage chicken breeding, using inspection robots instead of manual detection can solve the problems of large workload and low detection efficiency. However, poor driving stability of an inspection robot will lead to low inspection accuracy. In order to ensure high efficiency, accuracy, and stability of detection, this study takes the stability of visual navigation for inspection robots as the optimization goal, and designs an efficient and accurate road extraction algorithm and a navigation line fitting algorithm with better robustness. The experimental results show that the algorithm proposed in this study can improve the stability of detection, achieve a faster running speed, and achieve a better detection effect and a higher detection efficiency while keeping the detection effect unchanged. The navigation algorithm can accelerate the automation process of large-scale cage chicken breeding and promote the realization of fast and accurate monitoring.

**Abstract:**

The speed and accuracy of navigation road extraction and driving stability affect the inspection accuracy of cage chicken coop inspection robots. In this paper, a new grayscale factor (4B-3R-2G) was proposed to achieve fast and accurate road extraction, and a navigation line fitting algorithm based on the road boundary features was proposed to improve the stability of the algorithm. The proposed grayscale factor achieved 92.918% segmentation accuracy, and the speed was six times faster than the deep learning model. The experimental results showed that at the speed of 0.348 m/s, the maximum deviation of the visual navigation was 4 cm, the average deviation was 1.561 cm, the maximum acceleration was 1.122 m/s^2^, and the average acceleration was 0.292 m/s^2^, with the detection number and accuracy increased by 21.125% and 1.228%, respectively. Compared with inertial navigation, visual navigation can significantly improve the navigation accuracy and stability of the inspection robot and lead to better inspection effects. The visual navigation system proposed in this paper has better driving stability, higher inspection efficiency, better inspection effect, and lower operating costs, which is of great significance to promote the automation process of large-scale cage chicken breeding and realize rapid and accurate monitoring.

## 1. Introduction

The inspection of cage chicken houses is usually used to collect chicken house data to evaluate the health status of chickens, which is an important component of precision breeding and intelligent breeding. Frequent and accurate chicken house inspections can obtain health information such as diet, water, feces, respiration, calling, mental state, exercise status, and egg laying, as well as environmental information such as temperature, humidity and ammonia concentration in the chicken house environment in a timely manner. Such information plays a key role in determining the health status of chickens and regulating the environment inside the chicken coop. The health status of chickens can be gathered through inspection, which can improve the management level and production efficiency of farms and ensure the quality, safety, and environmental protection of livestock and poultry products [1]. In order to ensure the effectiveness of inspections, the data collection process must be stable and accurate [2].

The cage chicken industry, as the most intensive breeding industry, has multiple rows and layers of metal chicken cages in each chicken coop. Each cage contains multiple chickens, equipped with automatic feeding lines and automatic manure cleaning belts. The inspection of chicken coops is mainly achieved through two methods, manual inspection and robot inspection, and the inspection of cage chicken coops still relies on manual inspection [3]. Manual inspection relies more on the work experience and subjective judgment of workers, with a large workload and low efficiency, which can easily lead to missed inspections and false detections. At the same time, there may be fallen feed, feces, feathers, etc., inside the chicken coop, which can have bad effects on the health of workers. Inspection robots can carry a large number of environmental sensors and detection cameras, and automatically complete inspections through pre-set routes [4]. Compared with manual inspection, robot inspection can greatly reduce labor costs and can also provide more accurate chicken health data, making it more economically feasible in automated and large-scale production.

Agricultural robots, such as automatic feeding robots, egg picking and sorting robots, milking robots, and manure cleaning robots, have been widely used in livestock and poultry production [5,6,7]. However, the application of robots to farms is still a relatively new concept, and research on related robots is limited. A French robotics company has developed an autonomous modular robot called Octopus, which automatically flips and ventilates the bedding in a flat poultry house through a mechanism installed in the front, enhancing the effectiveness of disinfectants, thereby reducing chicken mortality and improving welfare [8]. Vroegindeweij et al. have developed an autonomous floor egg collection robot based on Lidar and visual navigation, which recognizes eggs through cameras and uses Lidar to scan for obstacles, thus achieving avoidance of chickens and collecting floor eggs [9]. However, the navigation of Lidar relies on the reflection information of obstacles. ChickenBoy, a roof-hanging detection robot produced by FAROMATICS (Barcelona, Spain), can detect the air quality and equipment operation in the livestock and poultry houses by hanging on the track [10,11]. Li et al. designed a track-based closed pigsty detection robot, which uses track-mounted environmental sensors and detection cameras to monitor the pigsty [12]. The navigation using the track has poor flexibility and needs to be reformed in the livestock farm. Feng et al. designed a livestock house disinfection robot based on the “Magnet-RFID” navigation mode [13]. This navigation also needs to lay RFID tags in advance, and the reading of the tags is easily affected by the electromagnetic environment of the livestock house. Yang et al. proposed a multi-sensor navigation system for a cow-feeding robot based on encoders, IMU, and ultrasonic sensors [14]. The system relied on railings as reference objects and implemented automatic navigation operations for the feeding robot through a fuzzy adaptive Kalman filtering algorithm. Krul et al. used drones to visually construct map navigation in greenhouses and cattle farms [15]. This UAV navigation method does not require the transformation of livestock and poultry farms, but requires a wide space to build maps in advance and is difficult to operate, so it is not suitable for use in cage-house environments with narrow space.

However, existing products and technologies are not specifically developed for the application of inspecting cage chicken coops, resulting in large lateral deviation of navigation and acceleration, and poor inspection effectiveness. The closed cage chicken coop is an indoor environment with no windows for daylighting and is illuminated by special light bulbs. The ground is generally gray and white concrete, and the ground usually has fallen feed, feces, feathers, etc. There are multiple rows of multi-layer hollow mesh metal chicken cages installed in the house, with an aisle width of about 1.1 m and a length of 80 m or even longer, and the aisles are equipped with cross-mounted light bulbs, which are about 2.5 m apart. This makes it difficult to apply common navigation methods in cage chicken coops. Although magnetic navigation has high accuracy, it requires pre-embedding magnetic tracks or renovating chicken coops, which increases the construction cost of chicken coops. Mujilang Intelligent Technology Co., Ltd. in Fuzhou, China, has developed a layer chicken breeding robot named “Mujilang”. The robot navigates through the magnetic stripe laid on the ground, solving the problem of inconvenient embedded wires, but the problem of magnetic stripe damage caused by daily washing of the chicken house needs to be solved. The indoor environment causes navigation systems using satellite positioning to malfunction, and a large number of metal components can interfere with electromagnetic signals, resulting in significant errors in positioning methods based on radio frequency signals [16,17]. Han et al. used 3D Lidar (Velodyne, California, USA) and the improved K-means clustering algorithm to solve the problem that the center line was difficult to obtain due to the weak light intensity and narrow channel in the cage chicken house [18]. This method used multi-line Lidar as the sensor, which has high cost and requires pre-processing and classification of point cloud data before navigation line fitting, and the algorithm was relatively complex. The hollow mesh chicken coops and complex obstacles lead to poor accuracy of navigation methods based on obstacle distance perception [19,20]. At the same time, the long corridor environment with similar structures makes Lidar prone to scene degradation problems. Zhang et al. used a UNet semantic segmentation model for visual navigation and a self-made DRITag for positioning to solve the visual navigation in the low-light environment of cage chicken coops [21]. This study only studied the positioning mode and used costly processors and deep learning models in visual navigation, which was expensive, complex and time-consuming, so further research on the real-time performance of visual navigation is needed.

Compared with other navigation methods, vision navigation is low-cost, can acquire rich texture information, and has more mature processing algorithms (Table 1), which have been widely used in the automatic navigation of agricultural robots. Zhang et al. designed an intelligent paddy field weeding robot with visual navigation based on SUSAN corner points and an improved sequential clustering algorithm, which proved to be effective in complex environments [22]. Ma et al. proposed an autonomous navigation algorithm for a wolfberry orchard-picking robot based on visual cues and fuzzy control [23]. The algorithm used the white region features of tree trunks for navigation line fitting and had good robustness. Liang et al. proposed an algorithm combining edge detection and OTSU to determine the seedling column contour of wide and narrow rows of cotton crops, and used the least square navigation line fitting method, which could well adapt to the interference of missing seedlings and rutting printing [24]. Chen et al. achieved visual navigation of a greenhouse cucumber picking robot by increasing the R and B component constraints and then the G component factor to separate the cucumber plant from the soil, combined with the proposed improved Hough transform [25]. This greying factor is good for the segmentation of objects with large color differences but less satisfactory for the segmentation of objects with similar colors.

The cage chicken house inspection robot’s main task is to carry environmental sensors and detection cameras, and conduct inspections on both sides along the chicken cage in the aisle. Therefore, the inspection process must be smooth and maintain a stable distance between the detection camera and the chicken cage to reduce the impact of lateral deviation on the accuracy of poultry detection. It is still a relatively new research direction to apply visual navigation to cage chicken coop inspection robots.

The main objective of this study is to provide a stable and reliable visual navigation for cage chicken house inspection robots, to improve the effectiveness of chicken detection during inspection operations. The specific objectives are as follows: (1) Propose a new grayscale factor (4B-3R-2G) to enhance the color feature difference between roads and background to achieve fast and accurate road extraction, to solve the problem of extraction difficulties and low accuracy caused by the similarity of road and background colors. (2) Propose a navigation line fitting algorithm based on the left and right boundaries of roads to solve the problem of poor robustness of existing visual navigation line extraction algorithms in road images with voids and missing boundaries, and improve the stability of navigation line fitting algorithms. (3) Evaluate the stability of the visual navigation during driving and the impact of the navigation on the detection system through a series of tests.

## 2. Materials and Methods

### 2.1. Navigation Hardware System

**Figure 1:**
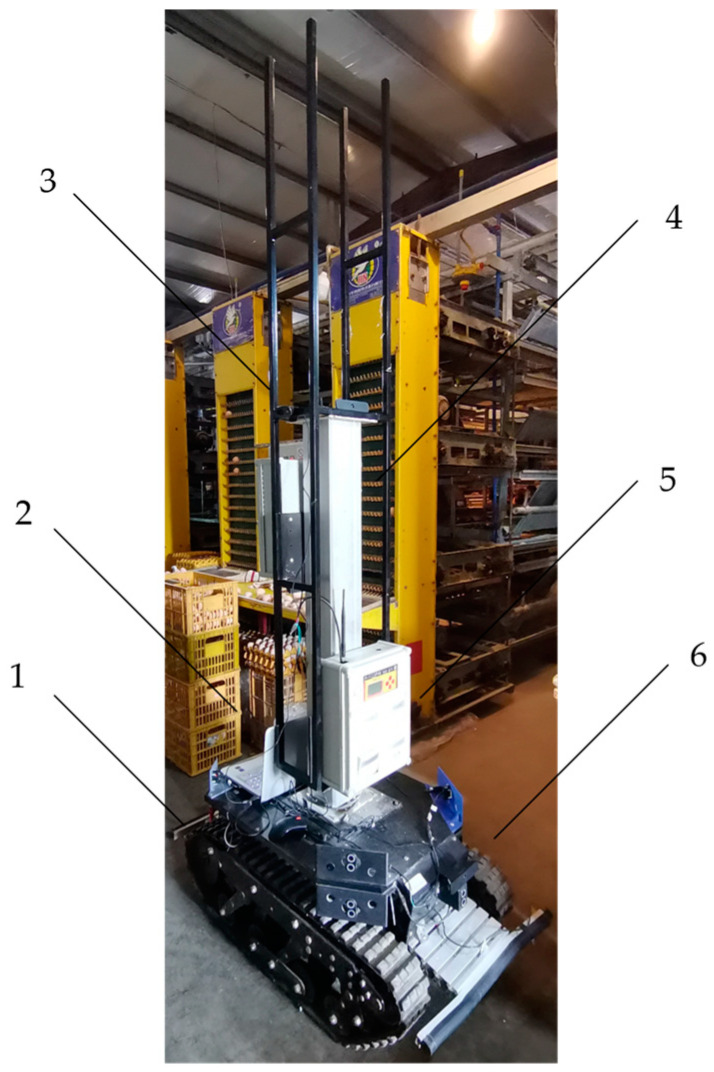
Schematic diagram of main components of a cage chicken coop inspection robot (1—track chassis; 2—PC; 3—monitoring cameras; 4—electric push rod; 5—environmental sensors; 6—visual navigation camera).

The inspection robot consists of a tracked robot chassis (Safari-600T, GUO XING INTELLGENT, Shandong, China), an inspection monitoring system, and a navigation system (Figure 1). The chassis of the tracked robot is equipped with a brushless motor, reducer, encoder, and motor controller. The brushless motor drives the tracks to provide power for the inspection robot. The steering action is achieved by the motor controller controlling the speed difference between the motors on both sides. The inspection and monitoring system includes a retractable electric push rod, environmental sensors, and monitoring cameras. The main indicators of robots are shown in Table 2.

**Figure 2:**
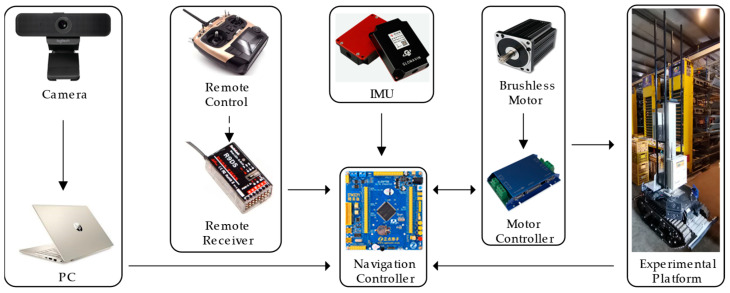
Overall framework of the navigation hardware system.

The navigation control system of the robot includes a camera, PC (Hewlett-Packard, Palo Alto, CA, USA), navigation controller (STM32F103ZET6, STMicroelectronics, Geneva, Switzerland), wireless receiver (R9DS, RadioLink, Shenzhen, China), IMU (A100, Glonavin, Hunan, China), and motor controller (GUO XING INTELLGENT, Shandong, China). The main hardware of the robot is shown in Figure 2. The camera model is Logitech C925E (Logitech, Newark, CA, USA), which is installed on the front of the robot, with a height of 45 cm above the ground and a downward tilt angle of about 8°. The main specifications of the camera are shown in Table 3.

The remote-control system consists of a wireless receiver and a remote control (R9D, RadioLink, Shenzhen, China) for switching between automatic and manual modes. The navigation controller needs to obtain the speed requirements and navigation path information of the inspection along the aisle for automatic navigation path tracking. The PC is a laptop equipped with an Intel i7-5500U CPU with 8G memory and a GTX 950M GPU. First, the PC on the inspection robot obtains the navigation path by processing the road image obtained by the camera, and transmits the data to the navigation controller through the USB. Then, the navigation controller calculates the pose of the visual navigation information as well as the lateral angle and encoder information, and sends the target speed instruction to the motor controller through the CAN bus. Finally, the motor controller adjusts the speed of the left and right wheels to reach the target speed, and feedbacks the real-time speed of the left and right wheels to the navigation controller through the CAN bus to realize the accurate navigation of the inspection robot along the aisle.

### 2.2. Robot Kinematics Model

**Figure 3:**
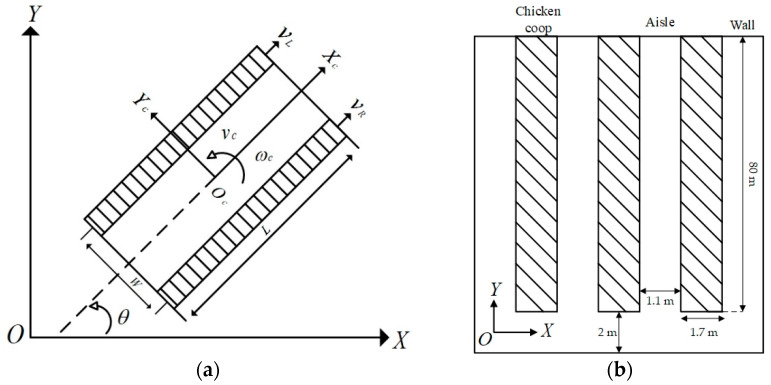
Robot local coordinates and global coordinates: (**a**) robot kinematics model; (**b**) global coordinates and coop layout.

To analyze the kinematics of the robot and describe the kinematics and state of the robot quantitatively, it is necessary to establish a kinematics model of the robot. In the analysis, the following simplifications and assumptions were made to the model: the center of the robot is at $O_{c}$; the robot moves only in one plane; slip is small enough to be neglected. The middle line of the leftmost chicken coop aisle was selected as the y axis of the global coordinate and the middle line of the transverse aisle as the x axis. The intersection of the *x* and *y* axes as the origin of the global coordinate and the direction is shown in Figure 3b. Figure 3a shows a plane motion model simplified based on the geometric structure diagram of the chassis of a tracked inspection robot and the coordinate system. Various movements of the robot are completed by controlling two driving wheels.

The robot state is represented by its center of mass $O_{c}$ and heading angle θ in the coordinate system (X,O,Y), where $v_{c}$ and $\omega_{c}$, respectively, represent the linear and angular speed of the tracked robot, $v_{L}$ and $v_{R}$, respectively, represent the traveling speed of the left and right driving wheels, L is the length of the track, and W is the axial distance between the driving wheels. According to the traveling speed of the left and right wheels of the tracked robot, the linear velocity $v_{c}$ and angular velocity $\omega_{c}$ of the robot can be expressed by the following equation.

(1) $$\left\{\begin{array}{c} v_{c}=\frac{v_{R}+v_{L}}{2}\\ \omega_{c}=\frac{v_{R}-v_{L}}{W} \end{array}\right.$$

At the initial moment, the geometric center $O_{c}$ of the tracked robot coincides with the global coordinate system O, and the position reached at the time t can be expressed by the following equation.

(2) $$\left\{\begin{array}{c} x_{t}=\int_{0}^{t}v_{c}\cos\theta dt\\ y_{t}=\int_{0}^{t}v_{c}\sin\theta dt\\ \theta_{t}=\int_{0}^{t}\omega_{c}dt \end{array}\right.$$

In the above formula, $x_{t}$ and $y_{t}$ are the real-time position coordinates of the center of the tracked robot, and $\theta_{t}$ is the rotation angle between the local coordinate system and the global coordinate system of the tracked robot. Equation (2) can be written as Equation (3).

(3) $$\left[\begin{array}{c} \dot{x}\\ \dot{y}\\ \dot{\theta } \end{array}\right]=\left[\begin{array}{cc} \cos\theta & 0\\ \sin\theta & 0\\ 0 & 1 \end{array}\right]\left[\begin{array}{c} v_{c}\\ \omega_{c} \end{array}\right]$$

Substitute Equation (1) into (3) to get Equation (4).

(4) $$\left[\begin{array}{c} \dot{x}\\ \dot{y}\\ \dot{\theta } \end{array}\right]=\left[\begin{array}{cc} \cos\theta & 0\\ \sin\theta & 0\\ 0 & 1 \end{array}\right]\left[\begin{array}{c} \frac{v_{R}+v_{L}}{2}\\ \frac{v_{R}-v_{L}}{W} \end{array}\right]$$

Equation (4) can be understood as a control system composed of input variables, $\mu ={\left[v_{R},v_{L}\right]}^{T}$, and state variables, $X=\;{\left[x,y,\theta \right]}^{T}$. It can be seen from Equation (4) that the coordinates of the robot’s traveling position are determined by the rotational angular velocity and linear velocity. Under the given linear velocity, the robot’s traveling trajectory is controlled by controlling the angular velocity. The inspection robot realizes the calculation and positioning of the relative position of itself and the chicken coop through dead reckoning, and accurately navigates the inspection robot along the aisles by converting the visual navigation information into the speed of the left and right wheels.

### 2.3. Navigation Process

**Figure 4:**
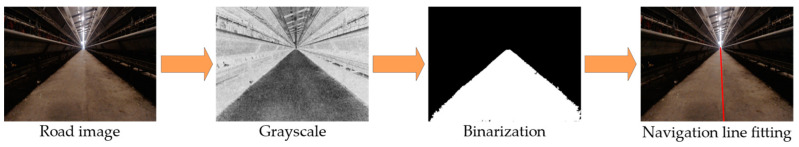
The overview of the navigation process.

The process of navigation along the aisle of a cage chicken house mainly includes the following three aspects: aisle image acquisition, road extraction, and navigation line fitting. The inspection robot first converts the images collected by the front camera into grayscale images through a grayscale factor, accelerating the image processing speed. Then, it binarizes the grayscale image and extracts it to the driving road. Finally, based on the features of the road boundary, the navigation line is calculated. The process of extracting navigation paths is shown in Figure 4.

#### 2.3.1. Aisle Image Acquisition

The test images were extracted from the video shooting at the aisle of the cage chicken coop at the Zengcheng Experimental Base of South China Agricultural University in Zengcheng District, Guangzhou City, Guangdong Province, China. The devices used are the Logitech C925E USB camera (Logitech, Newark, CA, USA) and HP laptop (Hewlett-Packard, Palo Alto, CA, USA).

#### 2.3.2. Road Extraction

In order to reduce the complexity of image processing and improve the speed and accuracy of image processing, grayscale images are generally used for road extraction. Murali et al. used the image entropy between the road and the background in the low-resolution gray image for navigation, which required a large difference in gray values between the road and the background [26]. Chen et al. used an improved excess green factor (2G-R-B) to increase the difference between green plants and soil, achieving the extraction of roads in greenhouses [25]. Lv et al. utilized color feature differences to extract apple images from fruit images in orchards using the R-G factor, and separated leaves from background using the 2G-R-B factor [27].

**Figure 5:**
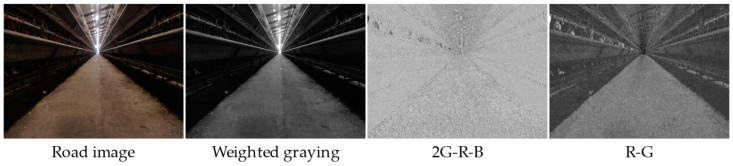
The effect of common grayscale.

In the cage chicken coop, the ground of the aisle is made of gray and white concrete, which is similar in color to the silvery white metal chicken coop in the background. After using the common grayscale factor, the difference between the road and the background is not significant, as shown in Figure 5.

**Figure 6:**
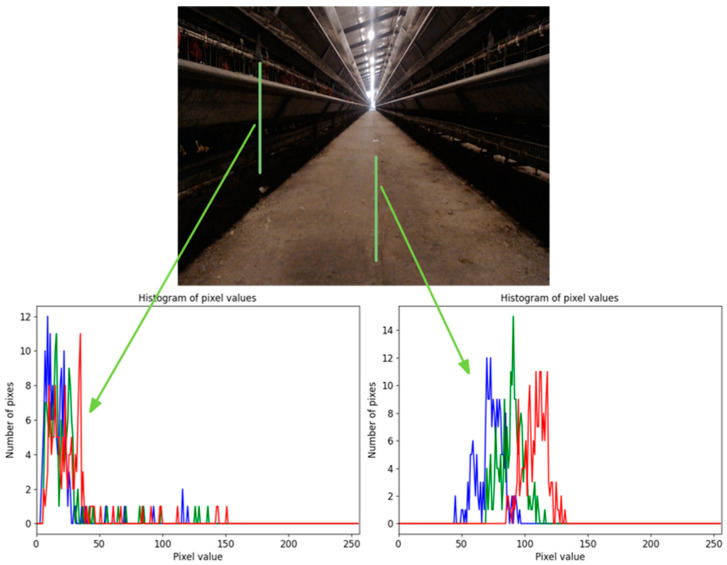
Navigation environment image analysis: the red, green and blue curves in the pixel distribution figures represent the number of red, green and blue channel pixels, respectively.

After using common grayscale factors for grayscale, the difference between the road and the background is small and difficult to accurately extract. The main reason is that these grayscale factors are not targeted at the environment of cage chicken coops, and cannot effectively separate the color differences between the road and the background. The aisle of a cage chicken house can be roughly divided into two areas: the road and the chicken cages on both sides. A vertical line between each road and background was drawn to better study the color feature differences between roads and backgrounds, as shown by the green lines in Figure 6. The pixel values of R, G, and B on these two lines are drawn in the corresponding coordinate system with red, green, and blue lines, respectively, and their distribution is observed. It can be seen from Figure 6 that the R, G, and B values of most pixels of the chicken coop are less than 25, and there is a small overlap with the R, G, and B values of road pixels. The R, G, and B values of road pixels are mostly in the range of 40–130. At the same time, the pixel values of the B channel presented by road pixels are smaller than those of the G channel, and the pixel values of the G channel are smaller than those of the R channel.

To strengthen the color feature difference between the road and the background, this paper improves the gray image conversion by increasing the value of the B channel and decreasing the values of R and G. Since the distribution range of the R, G, and B values of chicken coop and the distribution range of the aisle have a small overlap, the following grayscale factors are proposed after adjusting the weight values of the three channels:

(5) $$Gray_{0}(x,y)=4B(x,y)-3R(x,y)-2G(x,y)$$

R(x,y), G(x,y), and B(x,y) are, respectively, the values of the R, G, and B color channels corresponding to pixels with coordinates (x,y) in an RGB image, and Gray(x,y) is the gray value after using the grayscale factor proposed in this study.

In general, when grayscale factors are used, a fixed threshold is used to distinguish values outside the 0–255 range, replacing the normalization operation of the R, B, and G channels. Taking 2G-R-B as an example, the conversion equation is as follows:

(6) $$2G-R-B\left(x,y\right)=\left\{\begin{array}{cc} 2G-R-B(x,y), & 0\leq 2G-R-B(x,y)\leq 255\\ 255, & 2G-R-B(x,y)>255\\ 0 & 2G-R-B(x,y)<0 \end{array}\right.$$

The algorithm above will lose some road detail features, leading to a decrease in extraction accuracy. In order to improve the accuracy of road extraction and to avoid the gray scale range of the converted image not being within the range of 0–255, Equation (7) is used for normalization.

(7) $$Gray(x,y)=\frac{Gray_{0}(x,y)-Min_{Val}}{Max_{Val}-Min_{Val}}255$$

**Figure 7:**
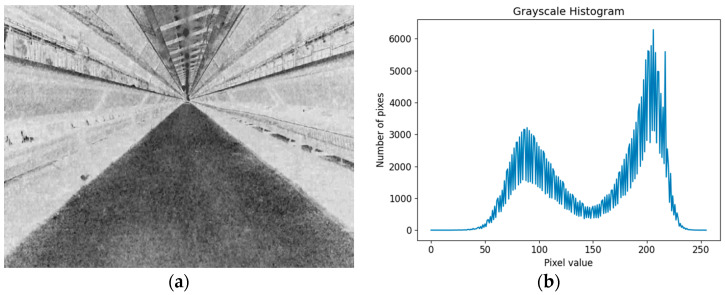
Grayscale effect: (**a**) road images after grayscale enhancement; (**b**) grayscale distribution histogram.

$Min_{Val}$ and $Max_{Val}$ are the minimum and maximum values of the gray value in the gray image before normalization, and Gray(x,y) is the gray value corresponding to the pixel with coordinate (x,y) after normalization. After using the grayscale factor proposed in this study, there is a significant difference in the grayscale values between the road and the background, and the grayscale histogram presents a bimodal image, as shown in Figure 7.

In order to maximize the difference between the road and the background image, and to have a certain anti-interference ability, a method based on inter-class variance is used for binarization. Assuming that there is a threshold value Th, all pixels of a grayscale image are divided into two categories: background $C_{1}$ (grayscale value less than Th) and road $C_{2}$ (grayscale value greater than Th). The mean values of these two categories of pixels are recorded as $m_{1}$ and $m_{2}$, the global mean value of the image is $m_{G}$, and the probabilities of pixels being divided into $C_{1}$ and $C_{2}$ are $p_{1}$ and $p_{2}$, respectively. The probability between them can be expressed by Equations (8) and (9).

(8) $$p_{1}m_{1}+p_{2}m_{2}=m_{G}$$

(9) $$p_{1}+p_{2}=1$$

The inter-class variance can be expressed as Equation (10) according to the definition of variance.

(10) $$\sigma^{2}=p_{1}{\left(m_{1}-m_{G}\right)}^{2}+p_{2}{\left(m_{2}-m_{G}\right)}^{2}$$

Equation (11) can be simplified by substituting Equation (8) into Equation (10).

(11) $$\sigma^{2}=p_{1}p_{2}{\left(m_{1}-m_{2}\right)}^{2}$$

**Figure 8:**
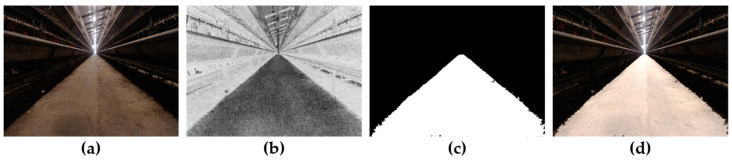
Navigation path extraction process: (**a**) road image; (**b**) image after grayscale enhancement; (**c**) extracted roads; (**d**) the fused image of the extracted road and the actual road.

Through traversing all gray values, when $\sigma^{2}$ is the maximum value ${\sigma^{2}}_{\;MAX}$, the corresponding TH is the optimal segmentation threshold $TH_{Best}$. Because the optimal segmentation threshold is selected from the image, a high segmentation accuracy can be obtained without the influence of road brightness and contrast. Figure 8 shows the process of extracting roads using the method proposed in this study. From Figure 8d, it can be seen that the extracted roads are basically consistent with the actual roads in the chicken house corridor, with high extraction accuracy.

#### 2.3.3. Navigation Line Fitting

In order to obtain accurate and fast navigation path, it is necessary to carry out navigation line fitting on the segmented road. Chen et al. used the midpoint Hough transform algorithm to fit the navigation paths of greenhouse tomato and cucumber spraying robots quickly and accurately [28]. The Hough transform is not sensitive to noise, occlusion, and other conditions, but the detection speed is slow and time-consuming. Ma et al. fitted the navigation path by dividing the image into upper and lower parts and using two different weights for weighted least squares to reduce the impact of low navigation reference crops [29]. Although the least square linear fitting method has good real-time performance, it is very easy to be disturbed by noise. Yu et al. used a polygon fitting method to extract navigation lines in the navigation of agricultural robots [30].However, the algorithm was complicated and its real-time performance was poor. Diao et al. utilized the vertical projection method to quickly and accurately locate the row feature points of corn crops and perform navigation line fitting [31]. The images after binarization will have holes and missing boundaries due to feces, feathers and feed on the ground, resulting in poor performance of the navigation line extraction method mentioned above when used in caged chicken coop.

**Figure 9:**
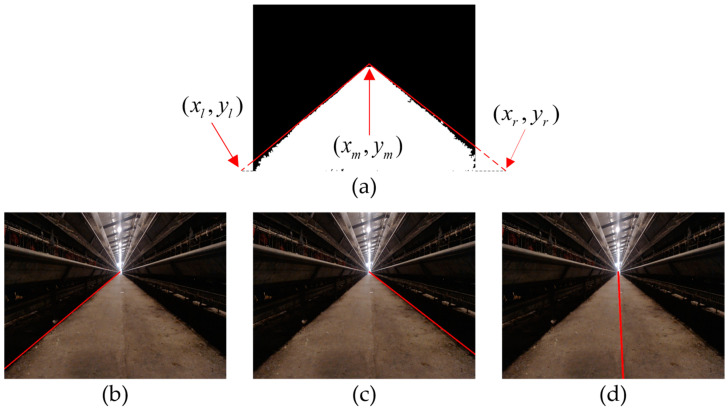
Road fitting results: (**a**) the left, middle, and right end points obtained by triangular fitting; (**b**) fitting effect of the left boundary under the road; (**c**) fitting effect of the right boundary under the road; (**d**) fitting effect of the navigation line under the road.

In this study, the chicken cages in the cage chicken house were arranged neatly, and the road boundaries were clearly visible on the images. To reduce the disturbance caused by image voids and missing boundaries on the navigation line fitting algorithm, and to improve the stability of the algorithm, a navigation line extraction method based on the left and right boundary features of roads was proposed. The specific steps are as follows:

(1) First, the road is fitted with the minimum outsourcing triangle, and the left end point $\left(x_{L},y_{L}\right)$, middle end point $\left(x_{M},y_{M}\right)$, and right end point $\left(x_{R},y_{R}\right)$ of the triangle are obtained, as shown in Figure 9a.

(2) The left boundary and the corresponding slope $k_{L}$ can be represented by Equation (12). The fitting effect of the left boundary under the road is shown in Figure 9b.

(12) $$\left\{\begin{array}{c} \frac{y-y_{M}}{y_{L}-y_{M}}=\frac{x-x_{M}}{x_{L}-x_{M}}\\ k_{L}=\frac{y_{L}-y_{M}}{x_{L}-x_{M}} \end{array}\right.$$

(3) The right boundary and the corresponding slope $k_{R}$ can be represented by Equation (13). The fitting effect of the right boundary under the road is shown in Figure 9c.

(13) $$\left\{\begin{array}{c} \frac{y-y_{M}}{y_{R}-y_{M}}=\frac{x-x_{M}}{x_{R}-x_{M}}\\ k_{R}=\frac{y_{R}-y_{M}}{x_{R}-x_{M}} \end{array}\right.$$

(4) The navigation line extracted according to the left and right boundaries can be written as Equation (14). The fitting effect of the navigation line under the road is shown in Figure 9d.

(14) $$y-y_{M}=\frac{k_{L}+k_{R}}{2}(x-x_{M})$$

**Figure 10:**
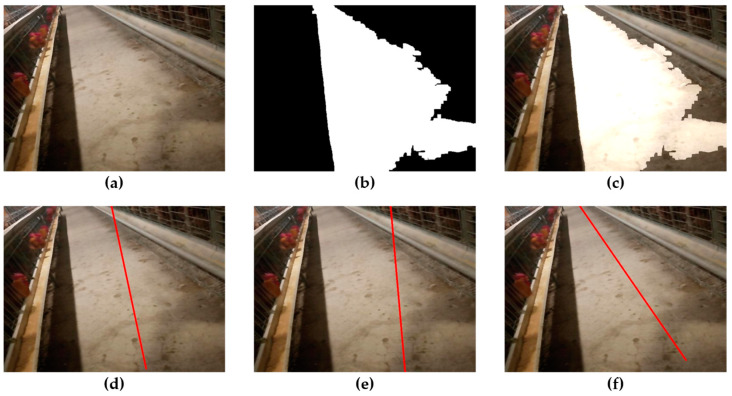
Results of different navigation line fitting algorithms: (**a**) road image; (**b**) extraction result; (**c**) fusion image; (**d**) least square fitting; (**e**) midpoint Hough transform; (**f**) fitting method based on boundary features.

The navigation line fitting algorithm proposed in this study can fit the navigation route well based on the characteristics of the left and right road boundaries (Figure 9b,c). Due to the presence of fallen feces, feed, and feathers and due to the camera shaking during walking in the hallway, the extracted roads inevitably have some missing parts, curved boundaries and internal voids in the image. This makes navigation line fitting methods such as midpoint Hough transform (Figure 10e) and least square fitting (Figure 10d) prone to errors and unstable fitting results. Compared with the midpoint Hough transform and least squares method, the navigation line fitting algorithm proposed in this study which fits the navigation lines based on the left and right boundary features of the road, still has a certain anti-interference ability and stable fitting effect in the above situations (Figure 10f).

### 2.4. Inspection Robot Performance Test

In this study, a series of tests and experiments were conducted to evaluate the accuracy and stability of the navigation system of inspection robots. Road segmentation is the foundation of visual navigation, and its accuracy and real-time performance affect the performance of navigation systems. Firstly, the accuracy and real-time performance of the road segmentation algorithm were tested, and the common road scene in the chicken house was used as the test picture. Then, the robustness of the navigation system was tested in a typical cage chicken house aisle to check the ability of the navigation system to correct different lateral and angular deviations. Finally, the stability was tested at different speeds, and the navigation system was comprehensively evaluated by measuring and analyzing lateral deviation and lateral acceleration, and by introducing monitoring camera detection number and accuracy as evaluation indicators.

#### 2.4.1. Road Segmentation Test

The accuracy and real-time performance of the road segmentation algorithm were tested by collecting multiple pictures of different lighting conditions (9:00 a.m.–11:00 a.m.), different aisle environments, and different shooting angles from cage chicken farms in Zengcheng Experimental Base of South China Agricultural University, Zengcheng District, Guangzhou, Guangdong, China. The test consisted of 100 pictures of a cage chicken house composite road, including a pure aisle, a person in the aisle, an obstacle in the aisle, a wall on one side of the aisle, and different colors of chickens on the left and right sides of the aisle. Pixel Accuracy (PA), Class Pixel Accuracy (CPA), Mean Pixel Accuracy (MPA), Intersection over Union (IoU), Mean Intersection over Union (MIoU) and time consumption were used to evaluate the performance of the algorithm. In addition to the 2G-R-B and R-G algorithms mentioned above, the semantic segmentation model was also compared.

The evaluation indexes are calculated as follows.

(15) $$PA=\frac{TP+TN}{TP+TN+FP+FN}$$

(16) $$CPA=PA_{classi}$$

(17) $$MPA=\frac{\sum_{i=1}^{n}CPA_{i}}{n}$$

TP is true positive; FP is false-positive; FN is false negative, and TN is true negative. PA represents the proportion of the total number of pixels for which the model predicts the class to be correct. CPA represents the accuracy of a certain category of pixels, where the accuracy of roads was calculated. MPA represents the proportion of pixels that are correctly classified for each category. In this article, there are two categories, so n=2.

(18) $$IoU=\frac{A_{r}\cap A_{p}}{A_{r}\cup A_{p}}$$

(19) $$MIoU=\frac{\sum_{i=1}^{n}IoU_{i}}{n}$$

$A_{r}$ represents the real pixel region, and $A_{p}$ represents the predicted pixel region. Generally, when IoU>0.5, the algorithm is successful in segmentation, and the closer the value of IoU to 1, the better the segmentation effect of the algorithm. MIoU shows the average of the intersection ratio of the model’s predictions for each category.

#### 2.4.2. Robustness Test

Therefore, the inspection robot should have certain robustness to adapt to the impact of the initial deviation. To verify the effects of different lateral and angular deviations on the robustness of the robot, a 20 m long aisle was selected in the cage chicken breeding farm of Zengcheng Experimental Base (9:00 a.m.–11:00 a.m.), South China Agricultural University, Zengcheng District, Guangzhou City, Guangdong Province, China. The robustness of initial pose lateral offsets of 10 cm and 15 cm and angle offsets of 5° and 10° were tested at 0.116 m/s.

#### 2.4.3. Navigation Accuracy and Stability Test

The inspection robot mainly provides services for poultry monitoring, and the stability of the navigation system directly affects the accuracy of poultry monitoring. Therefore, it is required that the robot has good centering driving ability, runs smoothly during the inspection process, and maintains a stable distance between the upper monitoring camera and the chicken cage, reducing the impact of lateral deviation changes on the monitoring camera. To analyze the performance of the navigation system under different speeds, a 20 m long aisle was selected in the cage chicken house of Lixing Agricultural Development Co., Ltd., Zhangxi Town, Raoping, Chaozhou, Guangdong Province, China (3:00 p.m.–5:00 p.m.), and tested at three speeds of 0.116 m/s, 0.232 m/s and 0.348 m/s. The inspection robot was equipped with infrared distance sensors on the side to record the lateral distance between the camera and the chicken cage. Meanwhile, the detection camera installed on top of the inspection robot monitors and identifies poultry during the testing process. The accuracy and driving stability of the navigation system were evaluated by the lateral distance deviation, the lateral acceleration, and the accuracy of poultry identification in the monitoring system, and compared with the inertial navigation using IMU and ultrasonic sensors.

## 3. Results and Discussion

### 3.1. Road Segmentation Test Result

**Figure 11:**
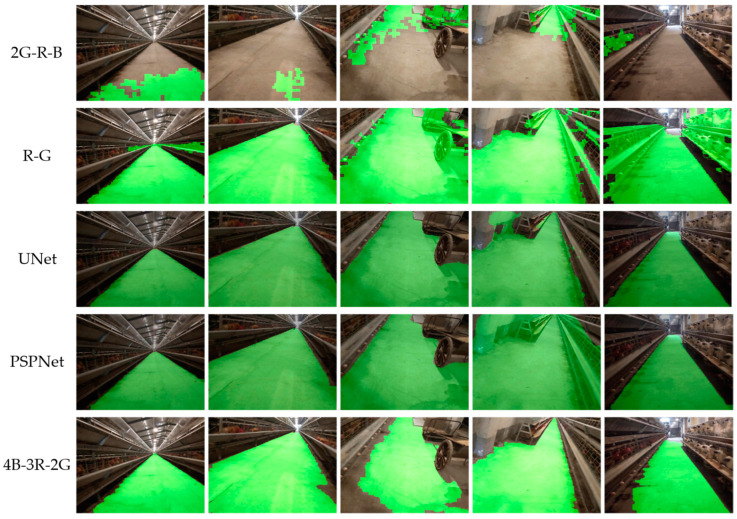
The segmentation effect of different algorithms.

The road segmentation effects of different algorithms are shown in Figure 11. It can be seen that the commonly used 2G-R-B and R-G algorithms in agricultural environments had poor segmentation performance on roads in cage chicken coops. However, the 4B-3R-2G had good segmentation performance in most scenarios inside cage chicken coops, especially in pure corridors. Compared with semantic segmentation algorithms, the 4B-3R-2G had lower segmentation accuracy in scenes with more obstacles than semantic segmentation algorithms. However, in the scenario where there was a chicken cage on one side and a wall on the other, the semantic segmentation algorithms made a significant error by dividing a portion of the wall into roads. Although the segmentation algorithm proposed in this study has a lower accuracy in this type of scenario, there is no obvious error, and the segmented roads are close to the actual situation.

In addition to segmentation accuracy, an algorithm’s time consumption is also a very important performance indicator, especially for inspection robots with lower performance. Although the accuracy of semantic segmentation algorithms has reached over 98%, the time consumption is generally above 110 ms (Table 4). Although deep learning methods have high accuracy in road segmentation, the complexity of the model structure makes it difficult to run them quickly on inspection robots with lower performance, and they are not suitable for navigation tasks with high real-time requirements, which restricts the further use of deep learning models. The segmentation algorithm proposed in this study, with similar accuracy, runs at a speed six times faster than the semantic segmentation model, making it very suitable for running on low-performance embedded devices [32]. The experimental results showed that the road segmentation algorithm proposed in this study achieves a segmentation accuracy of 92.918% for common scenes in cage chicken houses, with a time consumption of 16.448 ms. The algorithm has good segmentation performance and fast computational speed, meeting the navigation requirements of inspection robots.

### 3.2. Robustness Test Result

**Figure 12:**
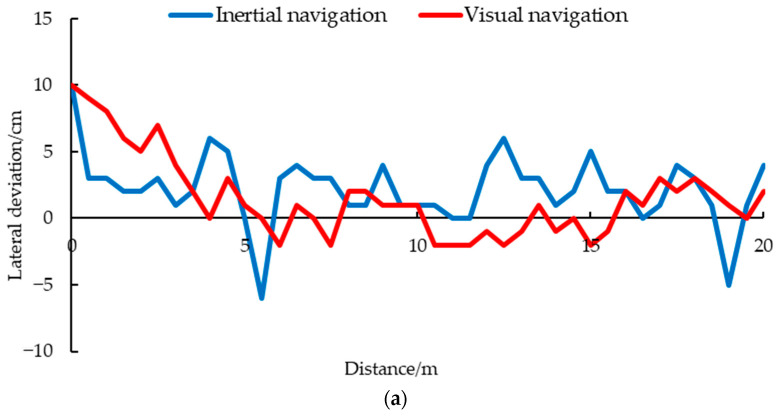

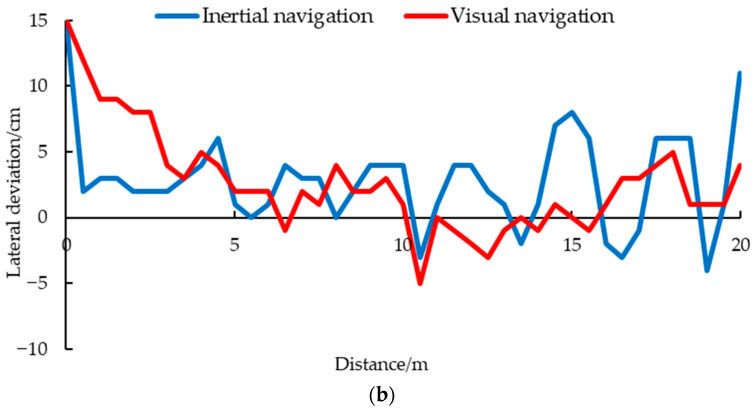
Navigation results with different initial lateral deviations: (**a**) 10 cm; (**b**) 15 cm.

From Figure 12, it can be seen that the inertial navigation using IMU and ultrasound corrected faster in the initial stage. This is because the initial lateral deviation is large, and the distance between the inspection robot and the chicken cage does not meet the set safety distance, so obstacle avoidance actions were carried out. If the distance between the inspection robot and the chicken cage after obstacle avoidance meets the set safety distance, it enters the inertial navigation stage. Because this navigation method cannot adjust the robot according to its own position, there will be a large navigation deviation in the inspection process. The inertial navigation had a maximum navigation deviation of 6 cm when the initial lateral deviation was 10 cm, and a maximum navigation deviation of 11 cm when the initial lateral deviation was set to 15 cm.

**Figure 13:**
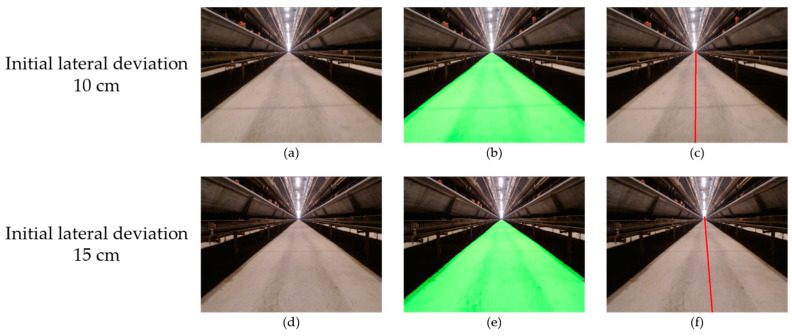
Visual navigation in the lateral deviation test: (**a**) road image with an initial lateral deviation of 10 cm; (**d**) toad image with an initial lateral deviation of 15 cm; (**b**,**e**) the segmentation results of (**a**,**d**); (**c**,**f**) the navigation line fitting results of (**a**,**d**).

When using visual navigation, whether the initial lateral deviation was to 10 cm or 15 cm, the deviation was not obvious in the image (Figure 13). The adjustment was relatively smooth, which was conducive to the stability of the detection camera image. When the initial lateral deviation was set to 10 cm, the maximum lateral deviation of visual navigation was 4 cm, and the maximum lateral deviation was 5 cm when the initial lateral deviation was set to 15 cm. Under different initial lateral deviations, the maximum lateral deviation of visual navigation did not change much, better than the inertial navigation.

**Figure 14:**
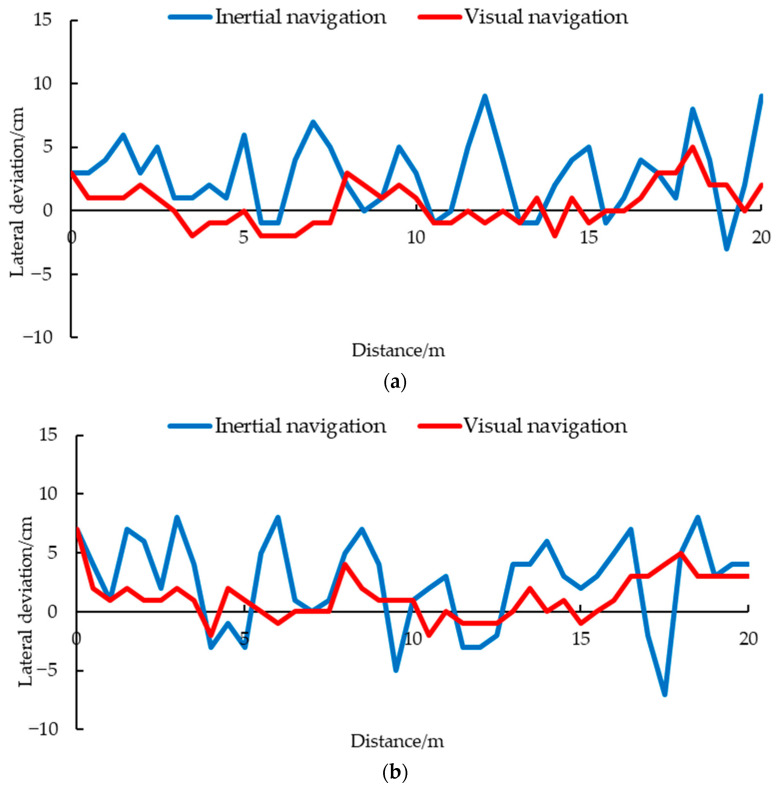
Navigation results with different angles of lateral deviations: (**a**) 5°; (**b**) 10°.

In the angle deviation test in Figure 14, there was a significant fluctuation in the lateral deviation of the inertial navigation. This is because in the initial stage, there is an angle deviation and the distance between the inspection robot and the chicken cage still meets the safety distance, so it is in the inertial navigation. When the distance between the inspection robot and the chicken cage is less than the safe distance after driving a certain distance, the obstacle avoidance action is triggered. After obstacle avoidance is completed, it enters the inertial navigation again. Due to the inertial navigation cannot perceive the initial deviation of the robot, the inspection robot constantly switches between obstacle avoidance and initial tracking angle, resulting in obvious multiple fluctuations in the lateral deviation. In the comparative experiment with an initial deviation of 5° and 10°, it was found that the larger the initial deviation, the greater the fluctuation amplitude of the lateral deviation in the inertial navigation.

**Figure 15:**
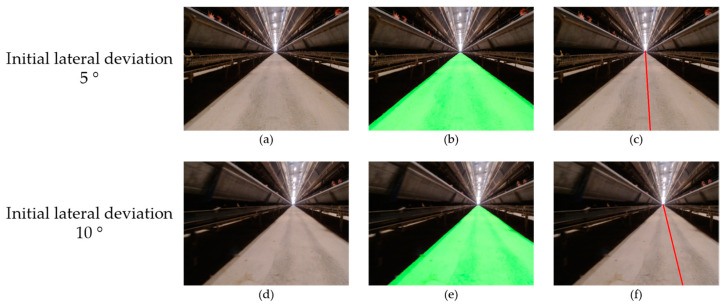
Visual navigation in the angle deviation test: (**a**) road image with an initial angle deviation of 5°; (**d**) road image with an initial angle deviation of 10°; (**b**,**e**) the segmentation results of (**a**,**d**); (**c**,**f**) the navigation line fitting results of (**a**,**d**).

The visual navigation line fitting method proposed in this study takes both sides of the chicken cage aisle as a reference, so the initial angle can be adjusted (Figure 15). Therefore, when there is an angle deviation, the inspection robot can adjust to it in a timely manner and keep the inspection process parallel to the aisle, ensuring that the monitoring camera can face the chickens directly. When the initial angle deviation of visual navigation was set to 5°, the maximum lateral deviation during driving was 5 cm, and the maximum lateral deviation was 5 cm when the initial angle deviation was set to 10°, which was smaller than the 9 cm and 8 cm of the inertial navigation. And the lateral deviation of visual navigation did not fluctuate much, which was significantly better than the inertial navigation. The robustness experiments showed that the navigation line fitting algorithm can quickly back to the set route, can operate stably under different lateral and angle deviations in the initial position, and has a certain degree of robustness.

### 3.3. Navigation Accuracy and Stability Test Result

**Figure 16:**
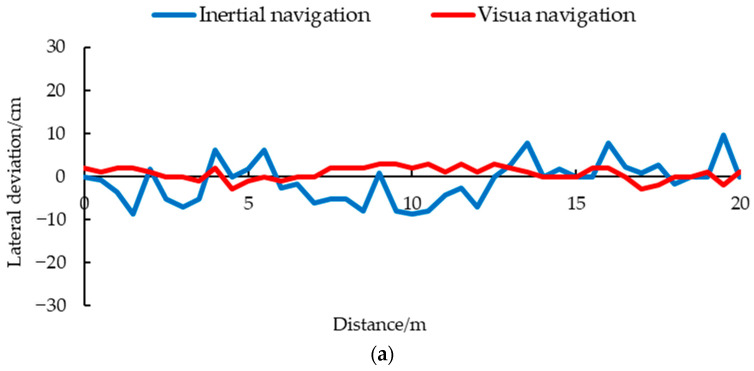

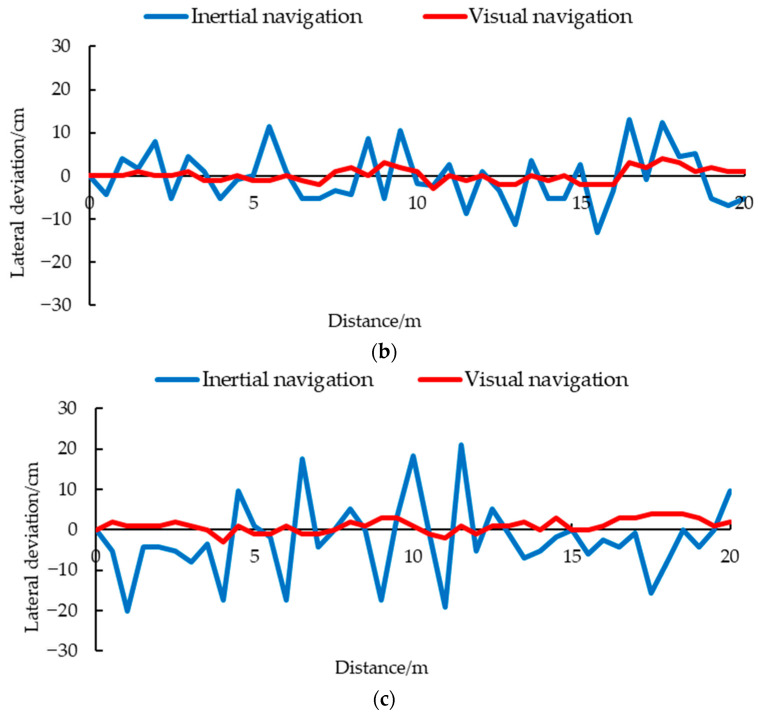
Navigation results under different speeds: (**a**) 0.116 m/s; (**b**) 0.232 m/s; (**c**) 0.348 m/s.

The results of different navigations under different driving speeds are shown in Figure 16. When the driving speed was set to 0.116 m/s, the maximum lateral deviation of visual navigation was 3 cm, and the average navigation deviation was 1.390 cm, while the maximum deviation of the inertial navigation was 9 cm, and the average deviation was 3.707 cm. At medium speed, the maximum deviation of visual navigation was 4 cm, with an average deviation of 1.220 cm. The maximum deviation of the inertial navigation was 13 cm, with an average deviation of 5.064 cm. As the driving speed increased, the lateral deviation also increased. When the driving speed was set to 0.348 m/s, the maximum lateral deviation of visual navigation was 4 cm, and the average lateral deviation was 1.561 cm, which was better than the maximum deviation of 21 cm and the average deviation of 6.902 cm in the inertial navigation. Compared with Vroegindeweij et al. using Lidar navigation, the visual navigation proposed in this study has higher accuracy [9]. 

Due to its high road segmentation accuracy, fast navigation line extraction speed, and good robustness, the maximum lateral deviation of visual navigation was 4 cm at high speeds (0.348 m/s), and the average time consumption was 20.275 ms. Compared with the algorithm proposed by Yu et al., the visual navigation has the advantage of reducing the fitting time of navigation lines in complex backgrounds [30].

**Figure 17:**
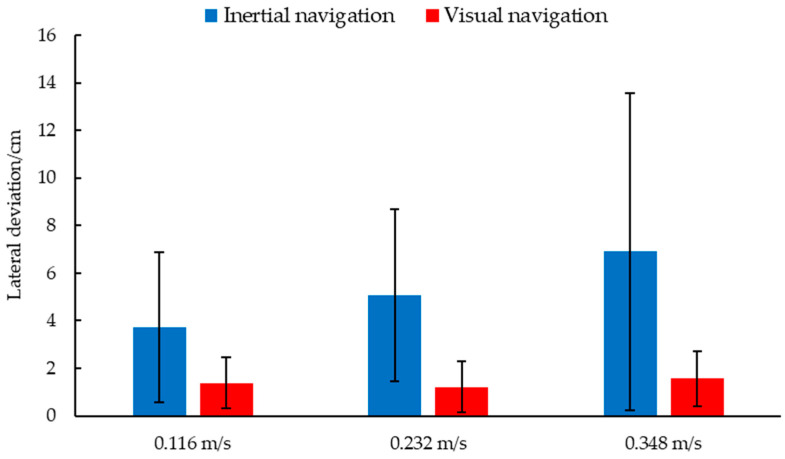
Lateral deviation and standard deviation of visual navigation and inertial navigation.

Figure 17 shows the lateral deviation and standard deviation of visual navigation and inertial navigation. Under the three test speeds, the average deviation and standard deviation of the visual navigation were smaller than those of the inertial navigation, and the navigation accuracy was higher. At the same time, taking inertial navigation as the control group, the *p*-values of visual navigation under the three speeds were all less than 0.001, indicating that visual navigation can significantly improve the navigation accuracy of inspection robots.

**Figure 18:**
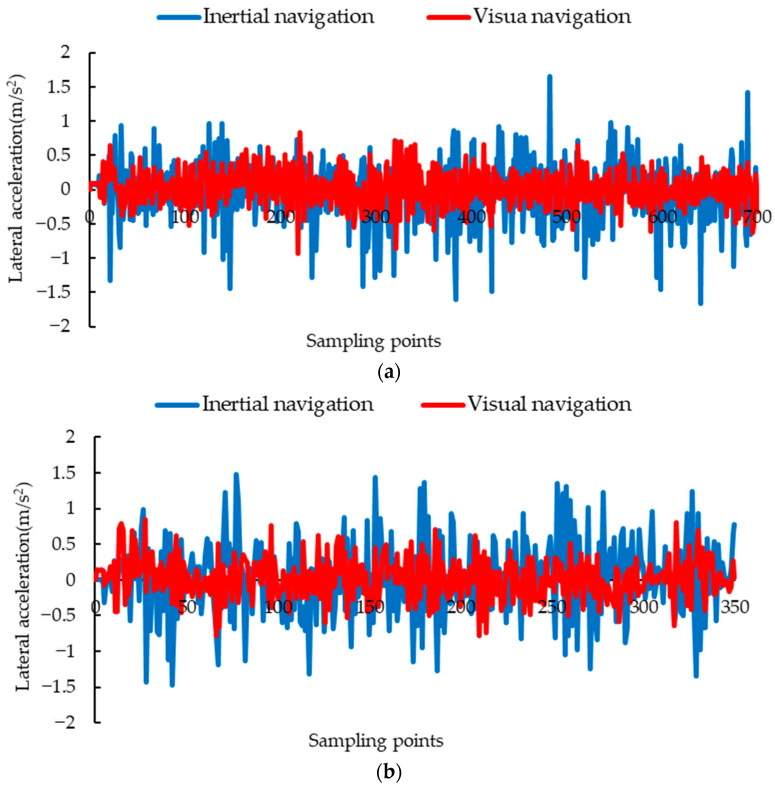

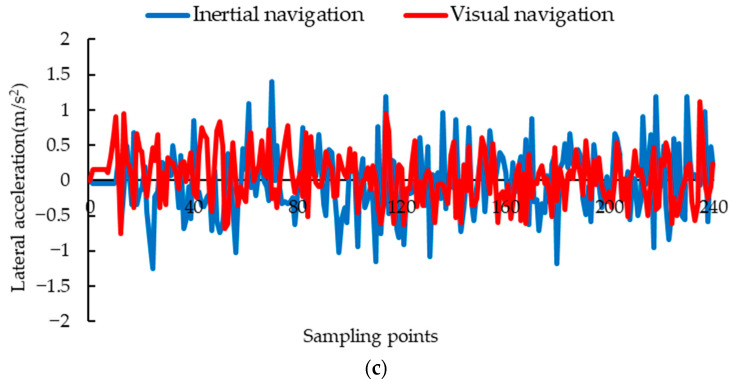
Lateral acceleration at different speeds: (**a**) 0.116 m/s; (**b**) 0.232 m/s; (**c**) 0.348 m/s.

Inertial navigation, using ultrasound and IMU, is limited by the principle of ultrasound sensors themselves. In this complex environment with a fence structure and uneven cross-section, it is easy to have unstable or even no echoes. The measurement data of ultrasonic sensors fluctuated greatly, leading to drastic adjustments and changes in the navigation system of the inspection robot, as shown in Figure 18.

When the driving speed was 0.116 m/s, the maximum lateral acceleration of visual navigation was 0.927 m/s^2^, the average acceleration was 0.189 m/s^2^, and those of the inertial navigation were 1.660 m/s^2^ and 0.300 m/s^2^ (Figure 18a). When the driving speed was 0.232 m/s, the maximum acceleration of the inertial navigation was 1.486 m/s^2^, the average acceleration was 0.418 m/s^2^, and those of the visual navigation were 0.844 m/s^2^ and 0.236 m/s^2^ (Figure 18b). Under the condition of high speed, 0.348 m/s, the maximum and average acceleration of visual navigation were 1.122 m/s^2^ and 0.292 m/s^2^, which were better than the 1.410 m/s^2^ and 0.367 m/s^2^ of the inertial navigation (Figure 18c). As can be seen from Figure 16, the maximum and average lateral acceleration of the visual navigation were smaller than those of the inertial navigation at a high speed, and even better than the inertial navigation at a low speed 0.116 m/s. The experimental results showed that the visual navigation can effectively reduce the lateral acceleration changes during driving, and can increase the stability of driving at low, medium, and high speeds.

In addition to the navigation accuracy, the impact on the poultry monitoring camera is also an important evaluation index of the performance of the inspection robot navigation system. Therefore, this study also introduced two indicators, detection number and detection accuracy, to comprehensively evaluate the navigation system. The trained YOLOv7 poultry detection model was used to evaluate the impact of visual navigation and inertial navigation on poultry detection tasks under the same driving path and three different driving speeds [33].

**Figure 19:**
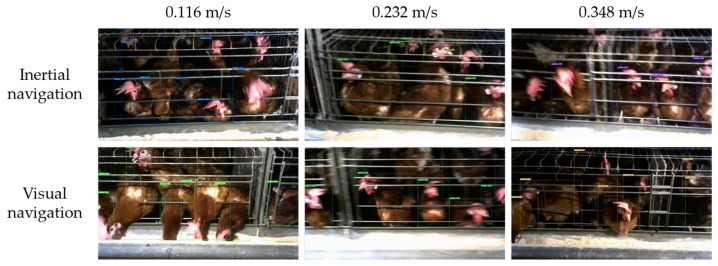
Detection results of different navigation methods at different speeds.

From Figure 19, the faster the navigation speed, the blurrier the chicken images captured by the monitoring system, and the lower the effective detection frequency and single recognition accuracy. For monitoring systems, the slower the inspection speed, the better the detection effect, but this will undoubtedly reduce the inspection efficiency [34]. At the same driving speed, the visual navigation method captured clearer images than the inertial navigation, with higher effective detection number and corresponding detection accuracy for a single image compared to the inertial navigation. The experimental results are shown in the Table 5.

Under the same inspection path, the average detection accuracy rate decreased as the inspection speed increased. The average detection accuracy rate of the visual navigation at high speed (0.348 m/s) was 1.097% lower than that at low speed (0.116 m/s), which was better than that of the inertial navigation (1.460%). This means that visual navigation stability is better, and the improvement of the inspection speed has a smaller impact on the poultry detection system. Thanks to the smaller lateral deviation and lateral acceleration fluctuations, the detection numbers of visual navigation were 43.787%, 23.514%, and 21.125% higher than those of the inertial navigation at three different speeds, and the detection accuracy was also improved by 0.865%, 0.346%, and 1.228%, respectively. At the same time, the inspection robot can use visual navigation to perform tasks at a faster speed with the same detection accuracy, achieving a faster working speed than the 0.24 m/s track inspection robot of Li et al. and one three times faster than Zhao et al.’s cage chicken inspection robot [12,35]. This shortens the single operation time and increases the number of inspections within a limited time, improving work efficiency. The experimental results showed that, compared with the inertial navigation, the visual navigation has better stability of the poultry detection camera, which can improve the detection number and accuracy, and that the poultry detection effect was better. At the same time, the inspection can be carried out at a faster speed to improve the operation efficiency.

## 4. Conclusions

As an important component of the cage chicken house inspection robot, the stability of the navigation system directly affects the detection results of the poultry detection camera. This study focuses on improving the accuracy and speed of road segmentation of visual navigation inspection robots and the robustness of navigation line fitting in complex scenes to improve the stability of navigation systems, so as to achieve better poultry detection results. A new grayscale factor (4B-3R-2G) was proposed to achieve fast and accurate road extraction, and a navigation line fitting algorithm based on the road boundary features was proposed to improve the stability of the algorithm. The proposed grayscale factor achieved 92.918% segmentation accuracy, the speed was six times faster than the deep learning model, and the lateral and angle deviations can be corrected to a certain extent. The experimental results showed that at a speed of 0.348 m/s, the maximum deviation of the visual navigation was 4 cm, the average deviation was 1.561 cm, the maximum acceleration was 1.122 m/s^2^, and the average acceleration was 0.292 m/s^2^, with the detection number and accuracy increased by 21.125% and 1.228%, respectively. Compared with inertial navigation, visual navigation can significantly improve the navigation accuracy and stability of the inspection robot and it has a better inspection effect. The visual navigation system proposed in this paper has a better driving stability, higher inspection efficiency, better inspection effect, and lower operating cost, which is of great significance to promote the automation process of large-scale cage chicken breeding and to realize rapid and accurate monitoring.

The visual navigation had good performance in testing, but there are still some errors in the above experiments, which are mainly caused by the following: (1) There is noise in the camera picture, which leads to slight fluctuations in the angle of the navigation line when the robot is stationary. (2) The vibration of the track itself and the camera vibration caused by the undulation of the road surface increases the error.

The method proposed in this study still has some limitations: the middle end point of the navigation line is not very accurate and the navigation line fitting effect needs to be improved when the angle deviation is particularly large.

Overall, the proposed method offered a high accuracy, low cost, and ease of implementation for the navigation of a cage chicken coop inspection robot. In the future, new methods will be explored to improve the navigation line fitting effect in the case of large angle deviation and achieve accurate positioning in this complex and electromagnetic interference environment.

## Figures and Tables

**Table 1:** Navigation technology in agriculture.

Navigation Method	Main Component	Advantage	Disadvantage
Track navigation	Track	High navigation stability	Structural renovation required, poor flexibility
Magnetic navigation	Magnetic stripes	Low cost, mature technology, and high navigation accuracy	Need to lay navigation landmarks, poor anti-interference ability, and poor flexibility of the route
Inertial navigation	Inertial sensor	High short-term accuracy, no need for external signals, good anti-interference ability	Low long-term accuracy and cumbersome initial calibration
Satellite navigation	Satellite positioning module	No cumulative positioning error, low cost	Cannot be used inside buildings, cannot be obstructed, and easily affected by the weather
Lidar navigation	Lidar	High resolution and high positioning accuracy	Poor detectability of smooth object surfaces and easy scene degradation in environments without obvious features
Visual navigation	Camera	Cheap, rich texture information, and the algorithm is mature	Large amount of information, time-consuming

**Table 2:** Specific parameters of crawler chassis.

Parameter	Performance
Dimensions (L × W × H)	1200 mm × 560 mm × 450 mm
Weights	136 kg
Ground clearance	100 mm
Rated power	650 W × 2
Running speed	0–1.16 m/s
Maximum load	100 kg
Motor rated voltage	48 V DC

**Table 3:** The main specifications of the camera.

Parameter	Value
Model	Logitech C925E
Dimensions (L × W × H)	126 mm × 45 mm × 73 mm
Weights	170 g
Resolution	1080 p/30 fps
DFOV	78°
Data interface	USB-A

**Table 4:** Accuracy and time consumption of different road segmentation algorithms.

Algorithm	PA	CPA	MPA	IoU	MIoU	Time (ms)
2G-R-B	47.469%	87.905%	49.213%	43.533%	26.523%	14.452
R-G	76.000%	80.220%	73.527%	64.291%	60.668%	14.001
UNet	98.146%	98.321%	98.289%	95.510%	96.186%	118.796
PSPNet	98.782%	98.492%	98.686%	96.849%	97.289%	113.462
4B-3R-2G	92.918%	95.160%	93.685%	85.549%	86.909%	16.448

**Table 5:** Results of poultry detection by inertial navigation and visual navigation at three speeds.

Navigation Mode	Speed	Detection Number *	Average Accuracy *
	0.116 m/s	11949	76.238%
Inertial navigation	0.232 m/s	5276	76.084%
	0.348 m/s	2807	74.778%
	0.116 m/s	17181	77.103%
Visual navigation	0.232 m/s	6898	76.430%
	0.348 m/s	3400	76.006%

* Detection number and average accuracy: detection number is the number of valid detection boxes in the detection system, and the average accuracy is the average accuracy of all valid detection boxes.

## Data Availability

The data presented in this study are available on request from the corresponding author.
